# Bee venom phospholipase A2 suppresses allergic airway inflammation in an ovalbumin‐induced asthma model through the induction of regulatory T cells

**DOI:** 10.1002/iid3.76

**Published:** 2015-08-09

**Authors:** Soojin Park, Hyunjung Baek, Kyung‐Hwa Jung, Gihyun Lee, Hyeonhoon Lee, Geun‐Hyung Kang, Gyeseok Lee, Hyunsu Bae

**Affiliations:** ^1^ Department of PhysiologyCollege of Korean MedicineKyung Hee University#1 Hoeki‐DongDongdaemoon‐GuSeoul130‐701Republic of Korea

**Keywords:** Asthma, bvPLA2, mannose receptor (CD206), regulatory T cells

## Abstract

Bee venom (BV) is one of the alternative medicines that have been widely used in the treatment of chronic inflammatory diseases. We previously demonstrated that BV induces immune tolerance by increasing the population of regulatory T cells (Tregs) in immune disorders. However, the major component and how it regulates the immune response have not been elucidated. We investigated whether bee venom phospholipase A2 (bvPLA2) exerts protective effects that are mediated via Tregs in OVA‐induced asthma model. bvPLA2 was administered by intraperitoneal injection into control and OVA‐challenged mice. The Treg population, total and differential bronchoalveolar lavage fluid (BALF) cell count, Th2 cytokines, and lung histological features were assessed. Treg depletion was used to determine the involvement of Treg migration and the reduction of asthmatic symptoms. The CD206‐dependence of bvPLA2‐treated suppression of airway inflammation was evaluated in OVA‐challenged CD206^‐/‐^ mice. The bvPLA2 treatment induced the Tregs and reduced the infiltration of inflammatory cells into the lung in the OVA‐challenged mice. Th2 cytokines in the bronchoalveolar lavage fluid (BALF) were reduced in bvPLA2‐treated mice. Although bvPLA2 suppressed the number of inflammatory cells after OVA challenge, these effects were not observed in Treg‐depleted mice. In addition, we investigated the involvement of CD206 in bvPLA2‐mediated immune tolerance in OVA‐induced asthma model. We observed a significant reduction in the levels of Th2 cytokines and inflammatory cells in the BALF of bvPLA2‐treated OVA‐induced mice but not in bvPLA2‐treated OVA‐induced CD206^‐/‐^ mice. These results demonstrated that bvPLA2 can mitigate airway inflammation by the induction of Tregs in an OVA‐induced asthma model.

## Introduction

The prevalence of allergic asthma is increasing in low and middle income countries 1. A recent report suggests that asthma currently affects more than 300 million people worldwide, and this number is likely to increase to 400 million individuals by 2025 2. Common symptoms of asthma include breathlessness, wheezing, chest tightness and coughing. Allergic asthma is a chronic immune disorder characterized by airway inflammation, airway hyperreactiveness (AHR), mucous hypersecretion, and excess production of T helper cell type 2 (Th2) cytokines 3, 4. Th2 immune responses have been implicated in the allergic responses of asthma through the production of IL‐4, IL‐5, and IL‐13, which leads to the production of allergen‐specific IgE from B cells and the recruitment of activated mast cells, basophils, and eosinophils into the airways 5, 6. It has been suggested that insufficient immunoregulatory properties lead to aberrant Th2‐mediated pulmonary inflammation.

Forkhead box P3‐expressing (Foxp3^+^) regulatory T cells (Tregs) are the most potent immunomodulators for the maintenance of immune homeostasis and exhibits various suppression and regulatory functions 7, 8. Deficiency of Tregs causes severe autoimmune diseases and chronic inflammation. Tregs inhibit the allergen‐specific Th1 and Th2 cell responses and therefore play important roles in the development of allergic disease 9. The adoptive transfer of CD4^+^CD25^+^ T cells into asthmatic mice reduces the eosinophil recruitment and Th2 cytokine production in the lung in an IL‐10‐dependent manner 10, 11. In contrast, Treg depletion enhances airway inflammation, as indicated by increased eosinophil recruitment and T cell proliferation 12.

Phospholipase A2 (PLA2) is an enzyme that catalyzes the hydrolysis of *sn*‐2 fatty acyl bond of membrane phospholipids to produce free fatty acids and lysophospholipids 13. PLA2 is a conserved component in multiple species and has been classified into three broad classes based on the cellular distribution: secreted (sPLA2), cytosolic (cPLA2) and Ca2^+^‐independent PLA2 (iPLA2). In addition to mammals, sPLA2 is present in the venoms of snakes, bees, cnidarian and scorpions. sPLA2 plays a central role in a wide range of cellular responses, including phospholipid metabolism, signal transduction and regulation of inflammatory and immune responses 14, 15, 16. Studies for sPLA2s have revealed their proinflammatory roles in mouse model of arthritis, asthma, or cardiovascular disorders 17, 18. Furthermore, bee venom PLA2 (bvPLA2), which is the major allergen in the venom, promotes anti‐venom Th2 cell response 19, 20. Otherwise, bee venom as an anti‐inflammatory drug has been used traditionally in the treatment of some immune‐related diseases, such as rheumatoid arthritis and multiple sclerosis 21, 22. Evidence of the involvement of distinct types of sPLA2 in anti‐inflammatory and immunosuppressive responses has been recently obtained 23, 24, 25. However, the efficacy and the mechanism of action of bvPLA2 involved in allergic asthma remain largely unknown.

Our recent study demonstrated that BV causes immune tolerance by increasing the population of CD4^+^CD25^+^Foxp3^+^ Tregs in lupus nephritis, cisplatin‐induced nephrotoxicity, and an allergic asthma model 26, 27, 28. In the present study, we investigated whether bvPLA2 induces immunosuppressive effects that are mediated via Treg cells in an OVA‐induced allergic asthma model. We also determined the role of CD206, which is a known receptor of bvPLA2, in the bvPLA2‐mediated suppression of airway inflammation. We found that treatment with bvPLA2 increased the Treg populations and suppressed the production of Th2 cytokines in the airway of OVA‐induced asthmatic mice. In addition, we showed that the bvPLA2‐mediated attenuation of asthmatic inflammation was dependent on the existence of CD206. Finally, this study has the potential to propose that bvPLA2 is a novel therapeutic target for controlling allergic asthma.

## Materials and Methods

### Animals

Male C57BL/6 mice (6∼7 weeks of age) were purchased from Charles River Korea (OrientBio, Sungnam, Korea). Foxp3^EGFP^Balb/c (C.Cg‐Foxp3^tm2Tch^/J) mice, Foxp3^EGFP^C57BL/6 mice and CD206^‐/‐^ mice (B6.129P2‐Mrc1^tm1Mnz^/J) were purchased from The Jackson Laboratory (Bar Harbor, ME). All of the mice were maintained under pathogen‐free conditions with air conditioning and a 12‐h light/12‐h dark cycle. All of the mice had free access to food and water during the experiments. The study was conducted in accordance with the Rules for Animal Care and the Guiding Principles for Animal Experiment Using Animals and were approved by the University of Kyung Hee Animal Care and Use Committee (KHUASP(SE)‐11‐025).

### Cell cultures

The spleens from Foxp3^EGFP^C57BL/6 mice were removed and disrupted over a cell strainer. After the red blood cells were lysed, the splenocytes were washed and resuspended in RPMI‐1640 (WelGENE INC., Taegu, Korea) supplemented with 10% fetal bovine serum (FBS), 50 IU/ml penicillin, and 50 ug/ml streptomycin (Hyclone, Logan, UT). Splenocytes were treated with various concentrations of PLA2 from honey bee venom (bvPLA2, *Apis mellifera*) (Sigma–Aldrich, St. Louis, MO) in the presence of plate‐bound anti‐CD3 (2.5 ug/ml) and soluble anti‐CD28 (10 ug/ml) (both from BD Biosciences, San Jose, CA). After 72 h, the cells were stained with fluorescently tagged anti‐CD4 and anti‐CD25 antibodies (eBiosciences, San Diego, CA) for flow cytometric analysis. All of the sample data were acquired on a flow cytometer (FACS Calibur, BD Biosciences) and analyzed using CellQuest Pro (BD Biosciences).

For the treatment of agonists for CD206, splenocytes were pretreated with mannan, zymosan, and MUC‐3 (10 and 100 ug/ml; Sigma–Aldrich). After 2 h incubation, the cells were treated with bvPLA2 for 72 h.

### OVA‐induced asthma animal model

The mice were sensitized through the intraperitoneal (i.p.) injection of 100 μg of ovalbumin (OVA; Sigma–Aldrich) combined with 20 mg of aluminum hydroxide in 100 μl of PBS on days 0 and 14. The mice were intratracheally (i.t.) challenged with 1% OVA in 50 μl of PBS six times between days 20 and 31. The negative‐control mice were sensitized and challenged with PBS alone. The bvPLA2 challenge groups were treated with an i.p. injection of 0.2 mg/kg bvPLA2 every three days from days 3–17. The experiments were performed 24 h after the last i.t. OVA challenge (day 32; Fig. 2A).

### Treg depletion

Foxp3^EGFP^Balb/c mice received a dose of 0.25 mg of rat anti‐mouse CD25 IgG (clone PC61.5) or total rat IgG on days 1, 8, and 15. The rat anti‐mouse CD25 IgG was generated in‐house from hybridomas obtained from the American Type Culture Collection (Manassas, VA). The efficacy of Treg depletion was analyzed by flow cytometry using PE‐anti‐mouse CD25 and fluorescein isothiocyanate (FITC)‐anti‐mouse CD4 antibodies.

### Analysis of bronchoalveolar lavage fluid (BALF)

The BAL fluid was collected by infusion and three extractions of 1 ml of ice‐cold PBS, and the lavages were pooled (mean volume, 2.0 ± ml). The recovered BAL fluid (70∼80%) was centrifuged at 1,300 rpm for 10 min. The total and differential cell counts of BAL fluid were determined using a hemocytometer and a cytospin preparation stained with Diff‐Quick (Life Technologies, Auckland, New Zealand). The BALF was centrifuged, and the supernatants were maintained at −80°C until cytokine analysis. The results are expressed as the total cell number ×10^4^.

### Assessment of cytokines in the BALF using an enzyme‐linked immunosorbent assay (ELISA)

The levels of IL‐4, IL‐5, IL‐13, and IFN‐γ in the BALF were assessed using a quantitative sandwich enzyme‐linked immunoassay kit (BD Biosciences for IL‐4, IL‐5, IFN‐γ, and R&D Systems, Minneapolis, MN, for IL‐13). A 96‐well‐plate was coated overnight at 4°C with anti‐mouse IL‐4, IL‐5, IFN‐γ, or IL‐13 mAbs in coating buffer. After washing, the wells were blocked with 5% FBS in PBS and 1% BSA in PBS for 1 h at 4°C and RT, respectively. Subsequently, the wells were loaded with 100 μl of BALF and incubated for 2 h at RT. After washing, the secondary peroxidase‐labeled biotinylated anti‐mouse IL‐4, IL‐5, IFN‐γ, or IL‐13 mAbs in assay diluents were added for 1 h. Finally, the plates were treated with TMB substrate solution (BD Biosciences) for 30 min, and the reaction was stopped by the addition of 50 μl of TMB stop solution per well. The optical density was measured at 450 nm using a microplate reader (SOFT max PRO, version 3.1. software, Sunnyvale, CA, USA). All of the results were normalized to the total amount of BALF protein in each sample.

### Determination of serum IgE and OVA‐specific IgE levels

The levels of serum IgE and OVA‐specific IgE were determined by ELISA. Blood was collected from the retro‐orbital plexus of mice while under ether anesthesia. Serum samples were obtained by centrifugation and stored at −20°C until assay. The plates were coated overnight at 4°C with anti‐mouse IgE antibody (BD Pharmingen) and anti‐mouse OVA IgE antibody (AbD Serotec, Oxford, UK). The serum samples were diluted 1:250 with 5% FBS in PBS (assay diluent), and the IgE levels were measured using a standardized sandwich ELISA according to the manufacturer's protocol. The optical density was measured at 450 nm using a microplate reader (SOFT max PRO, version 3.1. software).

### Measurement of airway hyperresponsiveness (AHR) with methacholine

On day 31, the animals were analyzed through non‐invasive lung functional measurement (All Medicus, Seoul, Korea) to assess the AHR. The mice were placed in a barometric plethysmographic chamber (All Medicus, Seoul, Korea), and baseline readings were obtained for 3 min. The enhanced pause (P_enh_) was calculated according to the manufacturer's protocol [i.e., (expiratory time/relaxation time‐1) × (peak expiratory flow/peak inspiratory flow)]. P_enh_ is a dimensionless parameter that represents a function of the proportion of the maximal inspiratory box pressure signals and a function of the timing of expiration. The results are expressed as the percentage increase in P_enh_ following challenge with the indicated concentration of methacholine (0, 25, 50, and 100 mg/ml).

### Histological examination

The lungs were removed, fixed in 4% paraformaldehyde (PFA), and embedded in paraffin after dehydration. The tissues were cut into 4‐µm‐thick sections and stained with hematoxylin‐eosin (H&E) for evaluation of inflammation and periodic acid‐Schiff (PAS) reagent for evaluation of goblet cells. The number of goblet cells within the bronchial epithelium was quantified as the percentage of PAS‐positive cells. Four bronchioles randomly selected from each section of mouse lung tissue were used to analyze, and the mean goblet cell coverage of each section was calculated 29, 30. The diameters of bronchi and bronchioles with goblet cell metaplasia were determined by using an Olympus BX51 microscope (Olympus, Tokyo, Japan) equipped with a DP71 digital camera (Olympus). For immunohistochemistry of myosin regulatory light polypeptide 9 (MYL9), the lung sections were treated with 0.3% H_2_O‐methanol for 20 min to block endogenous peroxidases. The sections were subsequently incubated with anti‐mouse MYL9 (1:50 dilution; Santa Cruz Biotechnology, Santa Cruz, CA), biotinylated anti‐goat IgG, and avidin‐peroxidase complex (Vectastatin ABC kit; Vector Laboratories, Burlingame, CA). The slides were developed with the peroxidase substrate 3'3‐diaminobenzidine tetrachloride (DAB; Zymed Laboratories, South San Francisco, CA). After immunohistochemical staining, the slides were counterstained with hematoxylin for 1 min and mounted with Canada Balsam (Show Chemical Co. Ltd., Tokyo, Japan). Four bronchioles were randomly selected from sections of the slides, and cross‐sections of lung parenchyma were captured, digitized and evaluated using Image Pro‐Plus 5.1 software (Media Cybernetics, Inc., Silver Spring, MD) 31, 32.

### Real‐time polymerase chain reaction

Total RNA from lung tissues was extracted using TRIzol reagent (Invitrogen, Carlsbad, CA) according to the manufacturer's procedure. cDNA synthesis was performed with Transcriptor First Strand cDNA Synthesis Kit (Roche, Indianapolis, IN). Real‐time quantitative PCR was conducted on LightCycler^R^ 480 (Roche), using 2× SYBR Green PCR master mix (Applied Biosystems, Foster City, CA). Mouse GAPDH was used to normalize sample amplification. The primers used were as follows: GAPDH (forward 5'‐TTC ACC ACC ATG GAG AAG GC‐3', and reverse 5'‐GGC ATG GAC TGT GGT CAT GA‐3'), sPLA2‐IID (forward 5'‐GGA GTC CCC TAG AAC CAA GC‐3', and reverse 5'‐CCG GAG CCT GAG CTA TTA TG‐3'), and sPLA2‐V (forward 5;‐TGG TTC CTG GCT TGC AGT GTG‐3', and reverse 5'‐TTC GCA GAT GAC TAG GCC ATT‐3')

### Statistical analysis

The statistical analyses of the data were conducted using the Prism 5 software (Graph Pad Software Inc., Sunnyvale, CA, USA). All of the values are presented as the means ± S.E.M (standard error of the mean). The differences between the means of the control and treatment samples were determined by one‐way ANOVA by the Turkey's Multiple Comparison test. Statistical significance was defined as *P* < 0.05.

## Results

### Effect of bvPLA2 on the induction of Tregs in the lung of OVA‐induced asthmatic mice

**Figure 1:**
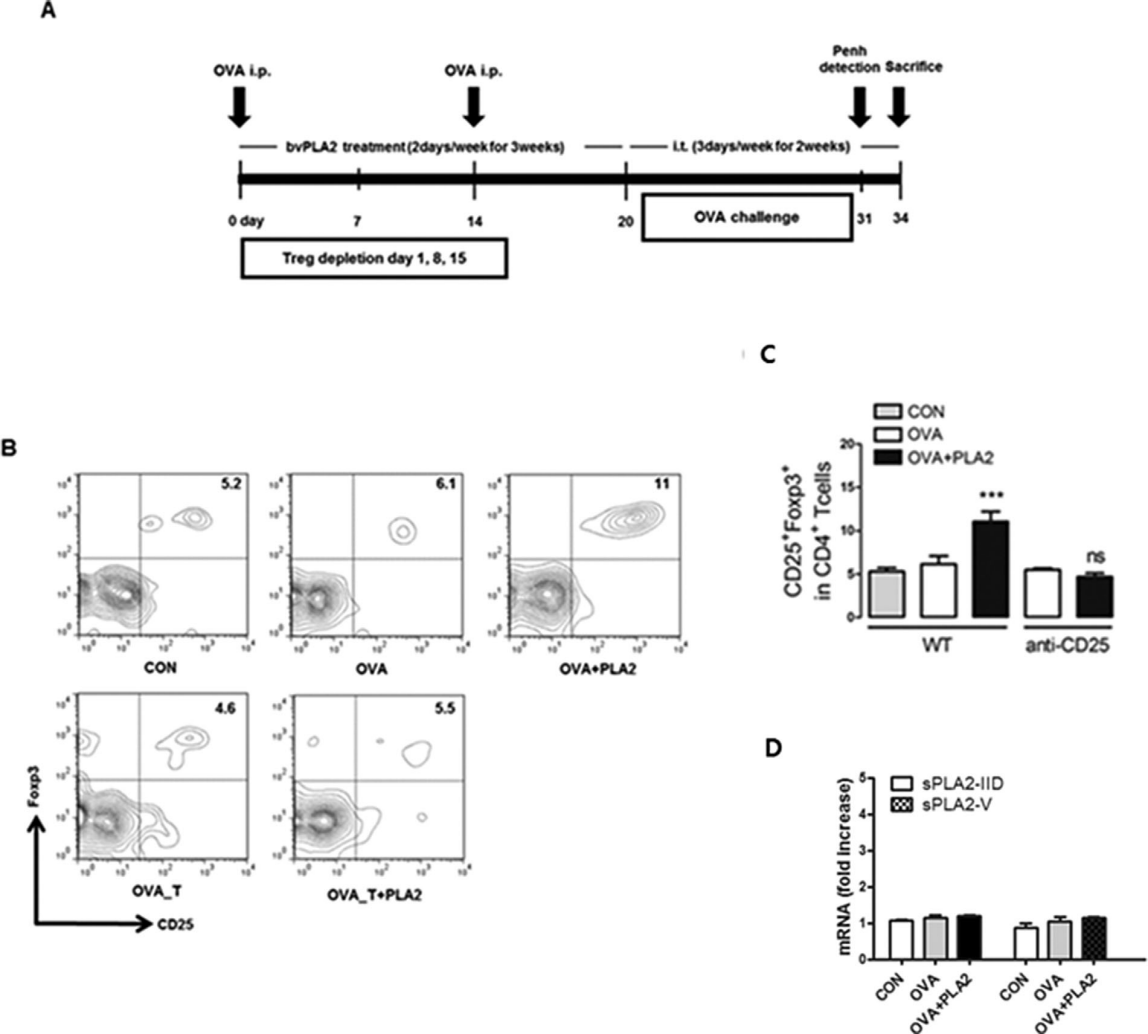
Effect of bvPLA2 on the induction of Tregs in the airway of OVA‐induced asthma mice. A: Schematic representation of the experimental protocol. Asthma was induced by the intraperitoneal injection (i.p.) of OVA combined with 20 mg of aluminum hydroxide on days 0 and 14. The mice were intratracheally challenged with 1% OVA in 50 μl of PBS six times between days 20 and 31. The control mice were sensitized and challenged with PBS alone. The bvPLA2‐challenged groups were treated with an i.p. injection of bvPLA2 every three days from days 3–17. On days 1, 8 and 15, 0.25 mg of rat anti‐CD25 IgG antibody was injected for Treg depletion. B: Pneumocytes were isolated from lung tissue, stained with anti‐CD4‐APC and anti‐CD25‐PE antibodies, and analyzed by flow cytometry. C: Percentages of CD4^+^CD25^+^Foxp3^+^ Tregs. D: Production of PLA2 was measured by Real‐time PCR. CON, PBS‐treated control group; OVA, OVA‐challenged group; OVA + PLA2, OVA‐challenged and PLA2‐treated group; OVA_T, OVA‐challenged and Treg‐depleted group; OVA_T+PLA2, OVA‐challenged, Treg‐depleted and PLA2‐treated group. The data are shown as the means ± SEM. Significance (*NS* > 0.05 *and* ***P < 0.001 vs. the CON group).

In the previous study, we found that the population of Foxp3^+^ Tregs was significantly increased in bvPLA2‐treated splenocytes comparing with the PBS‐treated cells in a dose‐dependent manner 33. Furthermore, recent reports have suggested that Tregs can suppress airway inflammation and hyperresponsiveness in an asthma model. To determine the involvement of bvPLA2 in the induction of Treg cells to the airway, the population of Treg cells in the lung tissue was determined in OVA‐induced allergic asthma mice. The mice were immunized twice with OVA in adjuvant on days 0 and 14. Six days after the second immunization, the mice were i.t. challenged with 1% OVA six times between days 20 and 31 (Fig. 1A). The Treg population in the lung tissue was not changed in the OVA‐challenged group (OVA group) compared with the control group (CON group), whereas the Treg cell population was markedly increased in the bvPLA2‐treated OVA‐challenged group (OVA + PLA2 group) (Figs. 1B and 1C). In contrast, the Treg population in the Treg‐depleted mice (OVA_T + PLA2 group) was not significantly altered by bvPLA2 treatment due to the effects of anti‐CD25 antibody injection. Next, to evaluated the secreted PLA2(sPLA2) production in OVA‐induced allergic asthma model, we analyzed the mRNA profile of lung tissues from WT, OVA, and OVA + PLA2 treated mice. There were no significant increase of sPLA2‐IID and sPLA2‐V in OVA‐induced and OVA + PLA2 mice compared with WT mice (Fig. 1D).

### Effects of bvPLA2 on lung inflammatory cells and levels of Th2 cytokine and serum IgE in OVA‐challenged asthma mice

**Figure 2:**
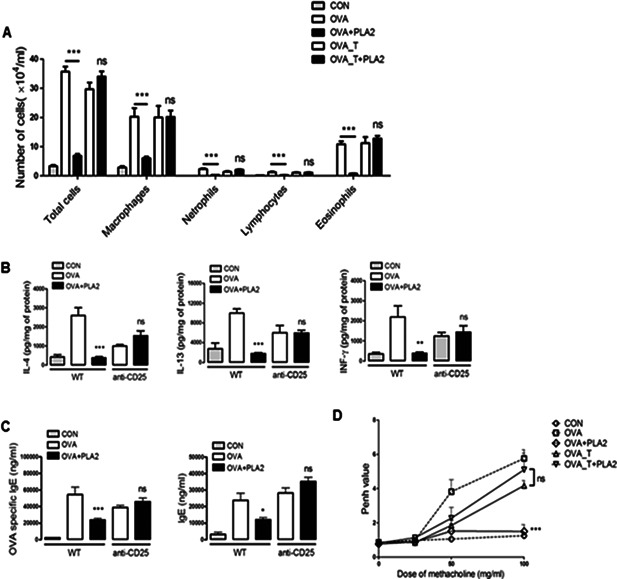
Effects of bvPLA2 on airway inflammation in OVA‐induced asthma mice. A: The total cells, eosinophils, macrophages, neutrophils and lymphocytes in the BAL fluid from the lung of mice were counted. B: The concentrations of IL‐4, IL‐5, and IL‐13 in the BALF were measured by sandwich ELISA. The expression of cytokines was analyzed in 100 μl of BALF and normalized to the total protein amount in each sample. C: The levels of total IgE and OVA‐specific IgE were evaluated by sandwich ELISA. D: Airway hyperresponsiveness was measured as Penh with increasing concentration of methacholine. CON, PBS‐treated control group; OVA, OVA‐challenged group; OVA + PLA2, OVA‐challenged and PLA2‐treated group; OVA_T, OVA‐challenged and Treg‐depleted group; OVA_T + PLA2, OVA‐challenged, Treg‐depleted and PLA2‐treated group. The data are shown as the means ± SEM. Significance (*P < 0.05, **P < 0.01, and ***P < 0.001 vs. the CON group).

The total cells, eosinophils, neutrophils, macrophages, and lymphocytes in the BAL fluid were increased by OVA challenge, which indicates that the induction of allergic asthma was successful. The increment of the inflammatory cell numbers was reduced by bvPLA2 treatment (Fig. 2A). Although bvPLA2 can suppress the inflammatory cells in OVA‐induced allergic asthma model, these effects were not observed in the Treg cell‐depleted groups. The level of Th2 cytokines was reduced by bvPLA2 treatment in OVA‐induced asthmatic mice (Fig. 2B). As shown in Fig. 2C, the serum IgE and OVA‐specific IgE concentrations were increased in the OVA group compared with the CON group, whereas the induction of the serum IgE level was diminished in the bvPLA2‐treated mice. However, increased Th2 cytokine and serum IgE levels were not observed following Treg depletion. Lung function was measured and expressed as enhanced pause (Penh) after methacholine exposures up to 100 mg/ml. The Penh values were increased in the OVA, OVA_T and OVA_T + PLA2 groups compared with the CON group. Treatment with bvPLA2 reduced the Penh values of the OVA group to the level observed in the CON group (Fig. 2D).

### Histopathological lung changes induced by bvPLA2 treatment in OVA‐challenged asthma mice

**Figure 3:**
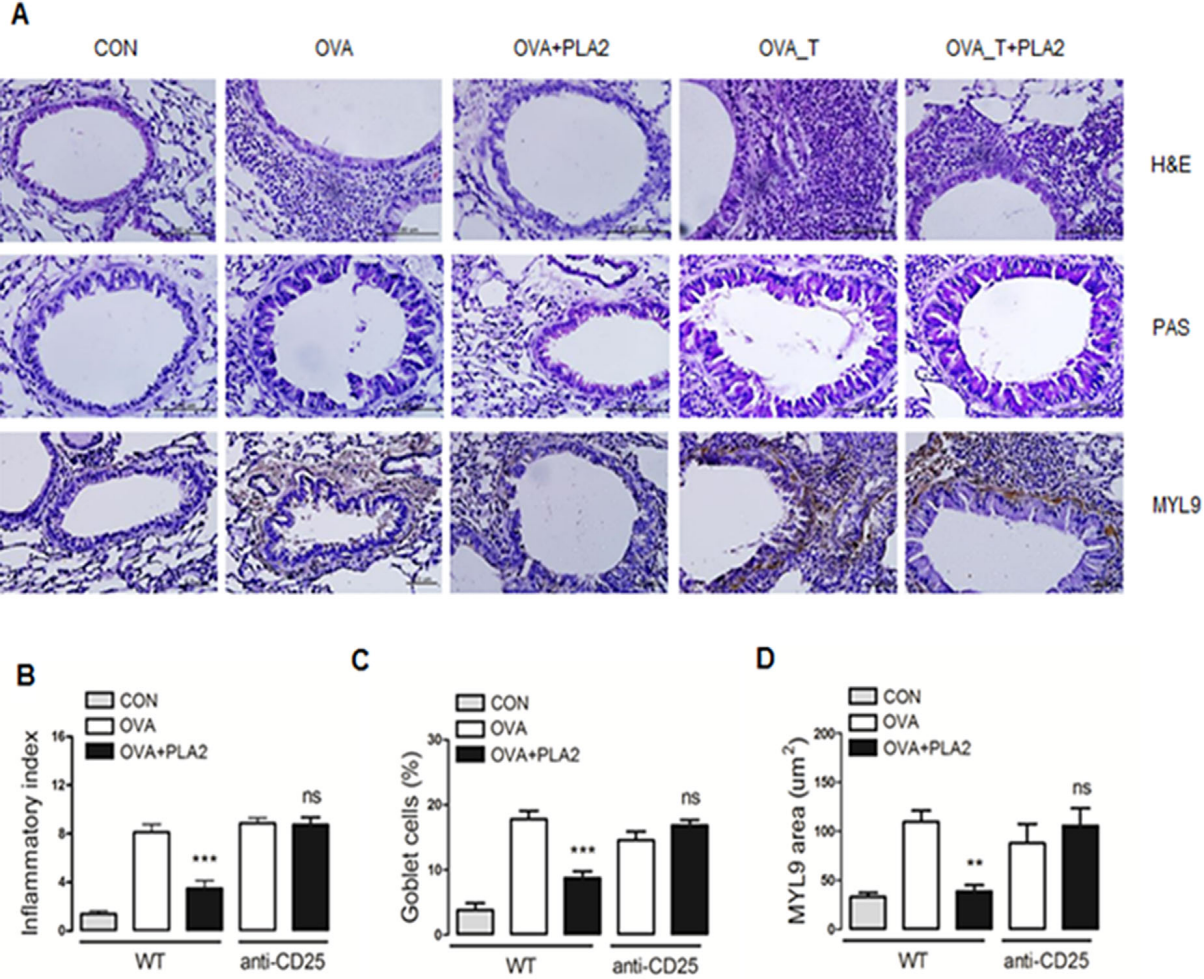
Effect of bvPLA2 treatment on histopathological changes in lung tissues during OVA‐induced allergic asthma. A: The lung tissues were stained with hematoxylin and eosin (H&E), periodic acid‐Schiff (PAS), and MYL9 antibody. Scale bars = 50 and 100 µm. B: The degree of inflammation was quantified using a semi‐quantitative scale. All of the randomly selected histological images were scored as the mean of inflammation index. C: PAS‐positive mucosal goblet cells around the bronchial airway were counted and are depicted as the percentage of goblet cells, as described in the Methods section. D: The thickness of smooth muscle was calculated based on the immunohistochemical images. CON, PBS‐treated control group; OVA, OVA‐challenged group; OVA + PLA2, OVA‐challenged and PLA2‐treated group; OVA_T, OVA‐challenged and Treg‐depleted group; OVA_T + PLA2, OVA‐challenged, Treg‐depleted and PLA2‐treated group. The data are shown as the means ± SEM. Significance (**P < 0.1, ***P < 0.001 vs. the CON group).

The histological analysis of lung tissues from mice that were exposed to OVA showed excessive infiltration of inflammatory cells into the lung parenchyma compared with the CON group (H&E panel in Figs. 3A). The bvPLA2 treatment markedly reduced the intense infiltration of inflammatory cells into the lung tissue (Fig. 3B). Consistent with the inhibitory effects on inflammatory cell infiltration into the lung, bvPLA2 treatment decreased the number of PAS‐positive goblet cells around the bronchial airway epithelium and the expression of myosin regulatory light polypeptide 9 (MYL9) in the peribronchial muscle layer of the lung. However, neither the number of PAS‐positive goblet cells nor the expression of MYL9 was altered by bvPLA2 treatment in Treg‐depleted mice (Figs. 3C and 3D).

These findings suggested that bvPLA2 has the potential to counteract allergic asthma‐associated airway inflammation and remodeling through the induction of Treg cells in the airway.

### CD206‐dependent role of allergic airway inflammation by bvPLA2 treatment in OVA‐induced asthma mice

**Figure 4:**
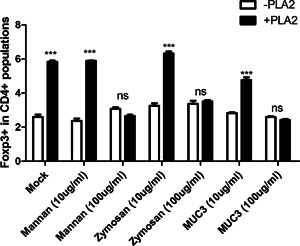
CD206‐dependent role of CD4^+^Foxp3^+^ Treg cells in vitro. The splenocytes were isolated from Foxp3^EGFP^C57BL/6 mice and pretreated with mannan, zymosan and MUC‐3 for 2 h before bvPLA2 treatment in the presence of anti‐CD3/CD28 antibodies. After 72 h, the cells were stained with anti‐CD4‐APC and analyzed by flow cytometry. The data are shown as the means ± SEM. The significance was determined by one‐way ANOVA followed by Newman‐Keuls multiple comparison test (***P < 0.001 vs. the –PLA2).

A previous study reported that bvPLA2 has high affinity with the macrophage mannose receptor CD206 34. First, we evaluated the requirement of CD206 for the induction of Treg population by bvPLA2 treatment. Agonists for CD206, mannan, zymosan and MUC‐3, were pretreated with bvPLA2. Treatment of mannan, zymosan and MUC‐3 significantly reduced the population of CD4^+^Foxp3^+^ Treg cells in the presence of bvPLA2 (Fig. 4). In addition, none of these CD206 agonist could induce Treg by itself.

**Figure 5:**
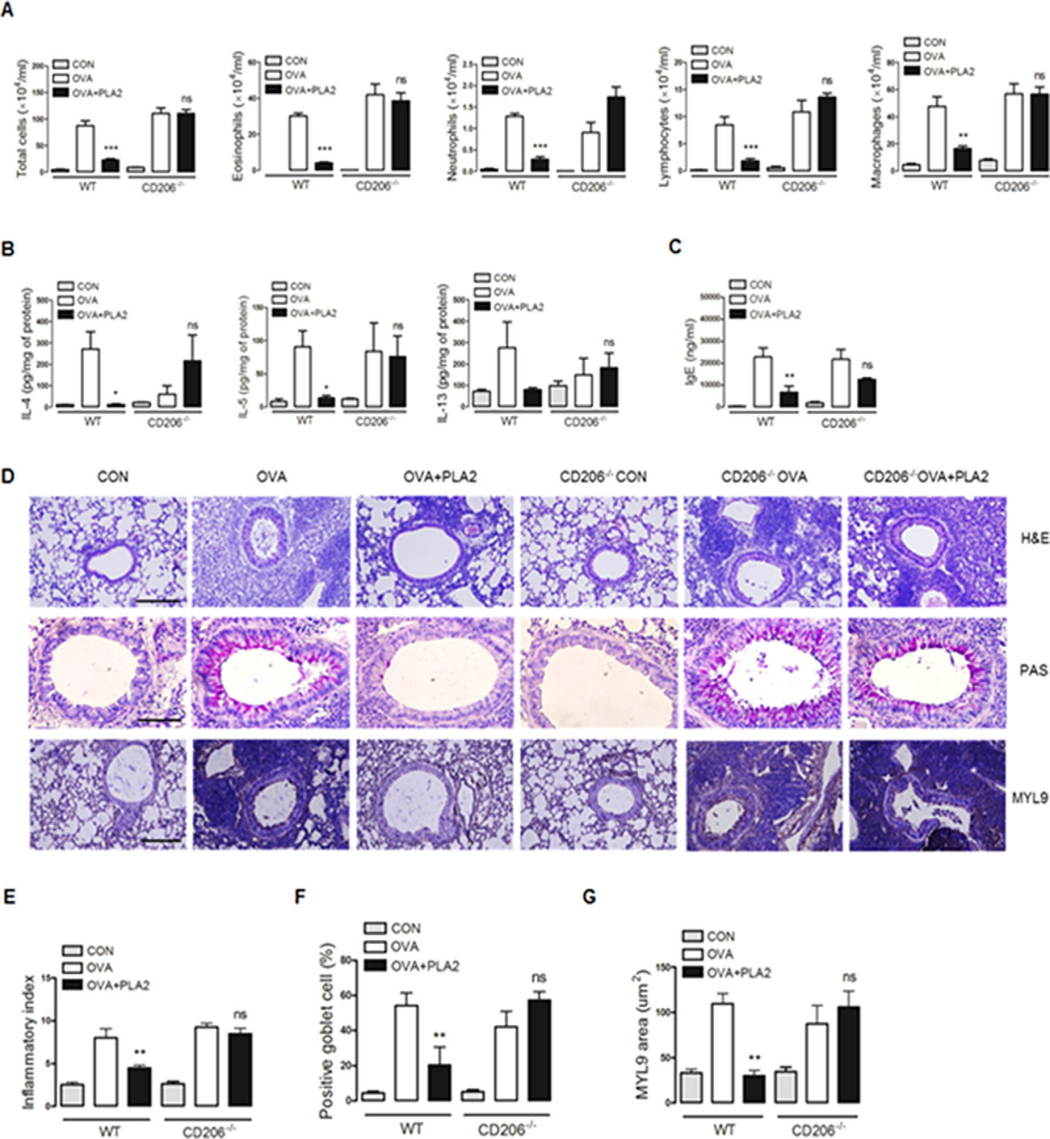
CD206‐dependent role of airway inflammation in response to bvPLA2 treatment in an OVA‐induced asthma model. PBS and OVA‐challenged C57BL/6 and CD206^‐/‐^ mice received an i.p. injection of 0.2 mg/kg bvPLA2. A: The total cells, eosinophils, neutrophils, macrophages and lymphocytes in the BALF were counted. B: The concentrations of IL‐4, IL‐5, and IL‐13 were measured using a sandwich ELISA kit. C: Blood samples were collected by cardiac puncture, and the levels of IgE in the samples were evaluated by ELISA. D: The lung sections were stained with H&E, PAS, and MYL9 antibody for analysis of cellular inflammation (original magnification ×200). E: The degree of inflammation was quantified using a semi‐quantitative scale. F: PAS‐positive mucosal goblet cells around the bronchial airway were counted and are depicted as the percentage of goblet cells. G: The thickness of smooth muscle was calculated based on the immunohistochemical images. CON, PBS‐treated control group; OVA, OVA‐challenged group; OVA + PLA2, OVA‐challenged and PLA2‐treated group; CD206^‐/‐^ CON, PBS‐treated CD206^‐/‐^ group; CD206^‐/‐^ OVA, OVA‐challenged CD206^‐/‐^ group; CD206^‐/‐^ OVA+PLA2, OVA‐challenged and PLA2‐treated CD206^‐/‐^ group. The data are shown as the means ± SEM. Significance (*P < 0.05 and ***P < 0.001 vs. the CON group).

The role of CD206 in the bvPLA2‐mediated modulation of airway inflammation was assessed in wild‐type C57BL/6 and CD206^‐/‐^ mice. The analysis of the BAL fluid from the OVA‐challenged WT and CD206^‐/‐^ mice revealed increased numbers of total cells, eosinophils, neutrophils, macrophages and lymphocytes in the airways, as determined by the differential cell BAL counts (Fig. 5A). Although the numbers of cells were markedly diminished in the OVA + PLA2 group, this effect was not observed in the CD206^‐/‐^OVA + PLA2 group. Reduction in the Th2 cytokine and serum IgE levels were found in allergic WT mice as a result of bvPLA2 treatment, but no significant alteration was observed in the allergic CD206^‐/‐^ mice (Figs. 5B and C). As determined through histological analysis, bvPLA2 decreased the inflammatory infiltration, PAS‐positive mucus‐secreting goblet cells, and MYL9‐positive cells compared with the OVA‐challenged group (Figs. 5D–G). The OVA‐challenged mice showed expression of MYL9 in the bronchial muscle layer of lung, and the treatment of bvPLA2 decreased the area of this smooth muscle layer (Figs. 5D and G). The allergic inflammation attenuated by bvPLA2 treatment was not detected in allergic CD206^‐/‐^ mice. These findings demonstrate that bvPLA2 has the potential to mitigate allergic asthma‐associated airway inflammation through interaction with CD206 in the airway.

## Discussion

In this study, we used different approaches to study the effects of bvPLA2 on regulatory T cells in an OVA‐induced allergic asthma model. Bee venom contains a variety of peptides and proteins, including melittin, phospholipase A2 (PLA2), adolapin, apamin, and mast cell degranulating peptide 13, 19, 35. PLA2 is one of the major components of BV that are commonly thought to play a significant role in inducing the allergic reaction associated with bee stings 19. However, the conflicting results in the literature indicate that BV can also exert anti‐inflammatory effects in various inflammatory reactions 36, 37, 38. Our group found that BV treatment ameliorates the clinical symptoms of asthma in mice, an effect that was associated with a significant increase in the Treg population 26.

We investigated the hypothesis that bvPLA2 can be a modulator of Treg populations in an OVA‐induced allergic asthma model. The in vitro results showed that the splenocytes treated with bvPLA2 exhibit an increased population of Tregs 33. Tregs and lung mast cells have been shown to produce secreted PLA2 39. We measured sPLA2‐IID and sPLA2‐V levels from the lung tissues in OVA‐induced allergic asthma mice. There were no significant increase of sPLA2‐IID and sPLA2‐V in OVA‐induced and OVA + PLA2 mice. Additionally, not only the infiltration of neutrophils and eosinophils but also the levels of IL‐4, IL‐5, and IL‐13 were reduced upon bvPLA2 treatment in OVA‐induced asthma mice. The serum total IgE and OVA‐specific IgE concentrations were also significantly decreased in the bvPLA2‐treated group. Interestingly, it has been reported that Tregs suppress IgE production via the direct regulation of B cells 40. The histological changes in the lung and airway inflammation paralleled the changes in the AHR to methacholine. In addition, increased levels of MYL9, which is the central regulator of the cellular contraction, have been found in the peribronchial muscle layers obtained from asthmatics 41, 42. However, the effects of bvPLA2 were abolished by Treg depletion; thus, this result strongly suggested that bvPLA2 can diminish allergic asthma inflammation by modulating Tregs.

Asthma is a complex disease that is characterized by reversible airway obstruction, elevated serum levels of IgE, airway eosinophilia, mucus hypersecretion and AHR to bronchospasmogenic stimuli 43, 44. According to a recent study, asthma may result from quantitative or functional deficiencies in the pulmonary Tregs that control Th1 and Th2 immune responses 45. Clinical advancement observed after allergen immunotherapy for allergic diseases, such as asthma, is associated with the induction of IL‐10‐ and TGF‐β‐producing Tr‐1 cells and Foxp3‐expressing IL‐10 T cells, which results in the suppression of Th2 cytokines 46.

Macrophage mannose receptor (MMR), which is also known as CD206, is a cell‐surface protein that belongs to a family of C‐type lectin receptors (CLRs), which also has other members, such as the sPLA2 M‐type receptor (PLA2R), the dendritic cell receptor DEC‐205 (CD205), and Endo180 (CD280) 47. CD206 is mainly expressed in macrophages and dendritic cells and appears to play a role in the early immune response against invading pathogens 48, 49. bvPLA2, which is structurally very similar to the snake venom and pancreatic PLA2s, is not recognized by the sPLA2 M‐type receptor (PLA2R). A high‐affinity and a specific binding site for bvPLA2 were found on the macrophage mannose receptor (CD206). Interestingly, the bvPLA2 binding sites are different from the binding sites for the snake venom and pancreatic PLA2s 34. To determine the role of CD206 in the response to treatment with bvPLA2 in OVA‐induced asthma mice, we investigated whether the bvPLA2‐mediated attenuation of allergic asthma could be correlated with CD206. The suppressive effect of bvPLA2 was abolished in OVA‐challenged CD206‐deficient mice. The induction of Th2 cytokines and eosinophil infiltration into the BALF was not recovered in the bvPLA2‐treated CD206^‐/‐^ mice.

Garg et al. 50 reported that Tregs were expanded by monocytes treated with mannose‐capped lipoarabinomannan (ManLAM) of *M. tuberculosis* and neutralizing antibodies to the mannose receptor (CD206), which binds ManLAM on antigen‐presenting cells, prevented the increase of Tregs. Furthermore, expansion of Tregs in response to M. tuberculosis depends on ManLAM and PGE2. PGE2 induces development of Tregs by Foxp3 gene expression and PGE2 acts through E‐prostanoid‐2 and ‐4 receptors to increase suppressor activity of Tregs 51, 52. We investigated the mechanisms of the regulation of Tregs via the binding between bvPLA2 and CD206. We found that bvPLA2 could not induce Tregs in co‐cultures of CD4^+^ T cells with macrophages. However, bvPLA2 significantly induced Tregs in co‐culture of CD4+ T cells with bone marrow derived‐ DCs [unpublished data]. This result indicates that although bvPLA2 is capable of binding to CD206 on DCs and macrophages, only DC can activate an immune‐suppressive program by bvPLA2.

In summary, we demonstrate the novel identification of bvPLA2 as an effective target for the regulation of Tregs in the airways, which results in the prevention of asthmatic symptoms. We also found that the bvPLA2‐induced Treg population to the lungs is correlated with CD206 to ameliorate allergic inflammation. Further studies aimed at refining the molecular mechanisms involved in the bvPLA2‐CD206 process could lead to the development of a new therapeutic strategy for the treatment of asthma patients.

## Conflict of Interest

The authors declare no commercial or financial conflict of interest.
